# Individual Insomnia Symptom and Increased Hazard Risk of Cardiocerebral Vascular Diseases: A Meta-Analysis

**DOI:** 10.3389/fpsyt.2021.654719

**Published:** 2021-05-14

**Authors:** Shiyu Hu, Tao Lan, Yang Wang, Lijie Ren

**Affiliations:** ^1^Neurology Department of Shenzhen Second People's Hospital, First Affiliated Hospital of Shenzhen University Health Science Center, Shenzhen, China; ^2^Spine Department of Shenzhen Second People's Hospital, First Affiliated Hospital of Shenzhen University Health Science Center, Shenzhen, China

**Keywords:** insomnia, symptom, cardiovascular disease, stroke, meta-analysis

## Abstract

**Objective:** Previous studies suggested that insomnia was associated with an increased risk of cardiocerebral vascular diseases (CVDs) but not clear in different insomnia symptoms. We performed a meta-analysis to investigate the association of individual insomnia symptoms and risk of CVDs.

**Methods:** In this meta-analysis, we systematically searched published articles by using electronic databases including PubMed, Cochrane Library, MedLine, and Google Scholar. Studies were enrolled if they indicated clear insomnia symptoms, prospective, and evaluated the association of insomnia symptoms and CVD outcome in adults free of CVDs at baseline.

**Results:** There were seven prospective cohort studies with sample sizes ranging from 2,960 to 487,200 included in this meta-analysis. Mean follow-up duration was 10.6 years. Insomnia symptoms of having difficulty initiating or maintaining sleep (DIS or DMS), non-restorative sleep (NRS), and early morning awakening (EMA) were analyzed in this study. All studies were compared under a random-effects model. NRS, DIS, and DMS were, respectively, related to 16% [hazard ratio (HR) 1.16, 95% CI 1.07–1.24], 22% (HR 1.22, 95% CI 1.06–1.40), and 14% (HR 1.14, 95% CI 1.02–1.27) higher risk of first-ever CVD incidence during the follow-up. Based on our analysis, EMA was not a risk factor of CVDs (HR 1.06, 95% CI 0.99–1.13).

**Conclusion:** This study suggested that symptoms of DIS, DIM, or NRS were associated with a higher risk of CVD incidence in insomnia patients free of CVDs at baseline. But this association was not significant in insomnia patients complaining about EMA.

## Introduction

Insomnia, defined as a person feeling of having difficulty initiating (DIS) or maintaining sleep (DMS) or feeling of non-restorative sleep (NRS), is the most common sleep disorder throughout the world, with an average prevalence of 33% (1–3). As a disease with high prevalence, insomnia could cause a health burden of an annual expenditure of 92.5–107.5 billion dollars years ago (4), which is a critical issue that needs to be solved.

There were about 35.7% of Chinese residents who complained about poor sleep quality in the research of China Chronic Disease and Risk Factor Surveillance (5). Even worse, insomnia has been proven to be associated with several ill health conditions, including hypertension, depression, diabetes, as well as neurological disorders (6–8). In recent years, some prospective studies indicated that insomnia disorder was associated with increased risks of cardiocerebral vascular diseases (CVDs) (9–12). However, the relationship of individual insomnia symptoms and risk of CVDs is still controversial because the inclusion criteria of insomnia patients in the published studies were not consistent.

DIS and DMS were identified as risk factors of CVDs, while early morning awakening (EMA) was associated with an increased risk in the study of Zheng et al. but insignificant in a Swedish cohort (13, 14).

Therefore, the aim of this study was to systematically review related prospective cohort studies and conduct a meta-analysis of all the available studies in order to investigate the association between different insomnia symptoms and the risk of first-ever CVDs.

## Methods

### Selection of Studies

We systematically searched published articles (through February 24, 2020) by using electronic databases including PubMed, Cochrane Library, MedLine, and Google Scholar. The keywords relating to insomnia as MeSH terms and text words (“Disorders of Initiating and Maintaining Sleep” or “Early Awakening” or “Insomnia” or “Insomnia Disorder” or “sleep complaints” or “sleep disturbance” or “Sleeplessness” or “poor sleep quality”) were used in combination with keywords relating to CVD (“myocardial infarction” or “myocardial ischemia” or “coronary artery disease” or “coronary heart disease” or “Cerebrovascular Accidents” or “stroke” or “cardiovascular disease” or “cardiocerebral vascular disease”). We limited our search to prospective cohort studies, supplemented by manually reviewing the reference of all retrieved papers. There was no language restriction of the studies. The inclusion criteria for the eligible articles were as follows: (1) the study should be prospective cohort or longitudinal cohort with follow-up; (2) patients with age >18 years, free of CVDs at baseline; (3) report specific insomnia symptoms and clear methods of sleep disorder assessment; (4) primary or secondary outcome should be the association of sleep disorder and CVDs; (5) the quantitative estimates of the univariate or multivariate adjusted hazard ratio (HR) and 95% confidence intervals (CIs) for CVD outcomes associated with insomnia symptoms should be provided. Relative risks (RRs) were considered equivalent to HR in the prospective cohort study; (6) the follow-up duration of each study should be longer than 2 years. Any study that did not meet the inclusion criteria was excluded. The articles were initially included or excluded based on title, abstract, and finally based on the complete article, decided by two independent interviewers.

### Data Extraction

The following data were extracted from the final included studies as the baseline characteristics: first author's name, year of publication, country, number of subjects at baseline, male gender proportion, mean age at baseline, insomnia symptoms of subjects, methods of insomnia disorder assessment, study outcome, follow-up duration, variables adjusted for confounding factors at the univariate or multivariate model. We also extracted HR and 95% CIs for CVD outcomes associated with insomnia symptoms for the statistical analysis.

### Statistical Analysis

The HR we used for analysis was from the most complete adjustment for confounders in the original study (adjusted confounders were shown in Table 1). All analyses used random-effects models, with heterogeneity assessed using the *I*^2^ statistic. Heterogeneity across studies was considered substantial if *I*^2^ > 50%. In order to identify the source of heterogeneity, we performed sensitivity analyses by eliminating each included study step by step and a subgroup analysis according to gender. Publication bias was evaluated by means of the Egger's test. We performed the statistical analysis by STATA version 15 (StataCorp LLC, College Station, Texas, USA), and statistical significance was considered when a two-tailed *p* < 0.05.

**Table 1:** Characteristics of enrolled cohort studies.

**References**	**Country**	**Subjects (*n*)**	**Male (%)**	**Age (years)**	**Insomnia symptoms**	**Insomnia assessment**	**Outcomes**	**Follow-up (years)**	**Adjusted factors**	**Quality score**
Zheng et al. (13)	China	4,87,200	40.90	51	DDF/DIMS/EMA	Self-reported insomnia symptoms for at least 3 d/wk. at baseline	IHD/acute MI/stroke	9.6	Age; education level; annual household income; marital status; alcohol consumption; smoking status; tea consumption; physical activity level; intake frequencies of red meat, fresh fruits and fresh vegetables; family history of heart attack and stroke; BMI; prevalent hypertension and diabetes mellitus at baseline; frequent use of sleep aid medications; frequency of snoring during sleep and depression or anxiety symptoms	5
Helbig et al. (15)	Germany	15,746	50.24	48.2	TFA/DSA	Self-report in a personal interview at baseline	Stroke	14	Age, educational level, physically active, alcohol consumption, current smoking activity, BMI, hypertension, diabetes, and dyslipidemia	5
Canivet et al. (14)	Sweden	13,617	43.10	45–69	DFA/Waking up during the night/waking up too early/Not feeling rested after sleeping	Instrument based on DSM-IV diagnostic criteria for insomnia	MI/stroke/ death due to IHD	13	Age	5
Laugsand et al. (16)	Norway	51,982	44.68	49.4	DIS/DMS/NRS	Self-administered questionnaire	AMI	11.4	Age, sex, marital status, education level, shift work, systolic blood pressure, total cholesterol, diabetes mellitus, BMI, physical activity, smoking, depression, and anxiety	5
Schwartz et al. (17)	USA	2,960	33.33	73	TFA/Trouble waking during night/Trouble waking too early/ Restless sleep	Four questions about insomniac complaints were asked at the baseline visit	MI	3	Age, gender, race, education level, number of prescription medications, self-rated health and depression	5
Meisinger et al. (18)	Germany	6,896	50.87	57.5	DIS/DMS	Two separate 3 category interview questions were asked	MI	10.1	Age, survey, BMI, education level, dyslipidemia, alcohol intake, parental history of MI, physical activity, regular smoking, hypertension, diabetes, and menopause status of women	5
Westerlund et al. (19)	Sweden	41,192	35.50	52.8	DFA/DMA/EMA/NRS	Sleep questionnaire included 13 items on sleep disturbances	MI/stroke//death from CVDs	13.2	Age, sex, education, employment status, smoking, alcohol, snoring, work schedule, depressive symptoms, self-rated health, physical activity, BMI, diabetes, lipid disturbance, and hypertension	5

*EMA, early morning awakening, also referred as trouble waking too early; NRS, non-restorative sleep, also referred as restless sleep or not feeling rested after sleeping; DIS, difficult initiate sleep, also referred as trouble falling asleep; DMS, difficult maintain sleep, also referred as waking up during the night, trouble waking during night, difficult staying asleep; TFA, trouble falling asleep; DSA, difficult staying asleep; DIMS, difficult initiate and maintain sleep; IHD, ischemic heart disease; MI, myocardial infarction; CVDs, cardiovascular diseases; AMI, acute myocardial infarction; BMI, body mass index*.

## Results

### Study Identification and Selection

**Figure 1:**
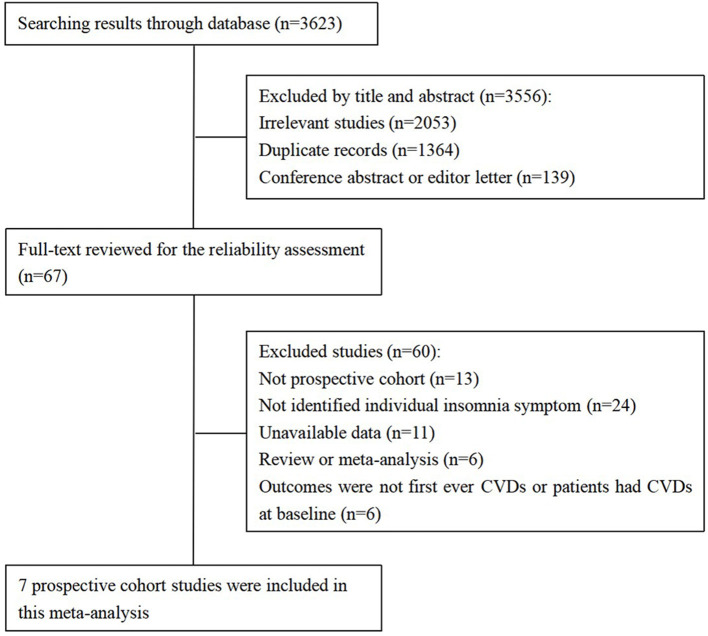
The flowchart of studies selection.

Based on our search strategy, there were a total of 3,623 records identified through the databases. We excluded 3,556 articles by title and abstract and then reviewed the full text to further exclude 60 studies according to the inclusion criteria of our study (follow chart shown in Figure 1).

Finally, there were seven prospective cohort studies included in this meta-analysis (13–19). Of these, there were three studies that separately analyzed the association between CVD outcome and different insomnia symptoms according to males and females, which means that they analyzed two outcomes in the same study. Therefore, we entered each of them as a single paper for the statistical analysis; as a result, there were 10 studies enrolled in the final analysis.

The entered articles included studies from China, USA, Germany, Norway, and Sweden, with sample sizes ranging from 2,960 to 487,200. The mean follow-up duration of this cohort was 10.6 years. As for the assessment of insomnia symptoms, two of the studies used the dichotomous variable (yes or no) (13, 15) while others used three to four category choices [(14, 16–19); e.g., never, occasionally, often, almost every night], in which cases we used the most serious category as the presence of insomnia symptoms based on the same criteria of the original articles. We evaluated the quality of these cohort studies by Newcastle–Ottawa Scale (NOS) (details shown in Table 1).

### Insomnia Symptoms and Cardiocerebral Vascular Outcomes

**Figure 2:**
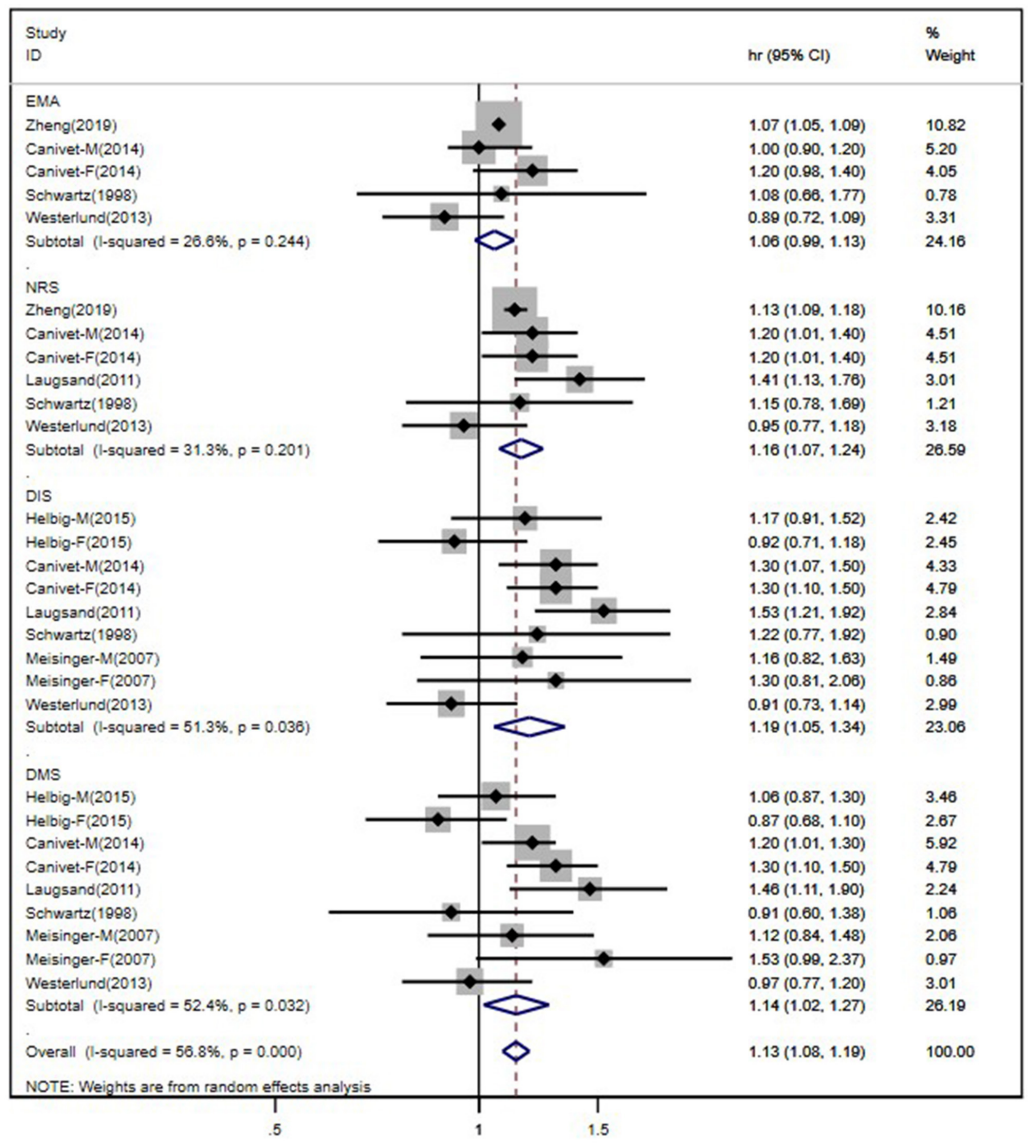
Forest plot of studies evaluating the association of individual insomnia symptoms and risk of cardiocerebral vascular disease.

After pooling all 10 studies' comparisons under a random-effects model, which were divided by different insomnia symptoms, the meta-analysis showed patients with NRS was in 16% higher risk of CVDs compared to those without corresponding symptoms (HR 1.16, 95% CI 1.07–1.24). Subjects who complained DIS and DMS had 22 and 14% increased risk of suffering CVDs, respectively, with respect to those without these two complaints (DIS: HR 1.22, 95% CI 1.06–1.40; DMS: HR 1.14, 95% CI 1.02–1.27). The association of EMA symptom and CVD incidence risk was not significant in data analysis (Figure 2). Overall, different insomnia symptoms were significantly associated with a 13% higher risk of suffering CVDs compared to subjects without any insomnia complaint (HR 1.13, 95% CI 1.08–1.19). There was no significant heterogeneity across the study of EMA and NRS, but a borderline heterogeneity was shown across the study of DIS and DMS. The overall heterogeneity was also significant with *I*^2^ = 56.8% (*p* < 0.001).

### Sensitivity Analyses and Publication Bias

As we had mentioned above, studies of patients with DIS and DMS symptoms reported borderline significant heterogeneity. Actually, according to our search strategy, the study of Eaker et al. (20) was also included at the beginning, but in our primary data analysis, the heterogeneity was much higher in the DIS studies (*I*^2^ = 62.5%, *p* = 0.04; after being excluded, *I*^2^ = 51.3%, *p* = 0.036). Therefore, we did not include this study in our final analysis. In order to figure out other possible difference across the studies, we performed a subgroup study based on gender for the sensitivity analysis. Since not all included studies had separate HR and 95% CI for CVDs and insomnia symptoms according to male and female, we only use the available data. The subgroup analysis based on the available studies showed that females with DIS and DMS symptoms reported much higher heterogeneity compared to males (Table 2). Egger's test was used to assess the publication bias of this study, which showed no significant results (*p* = 0.097).

**Table 2:** Subgroup analysis by genders.

	**Studies (*n*)**	**HR (95%CI)**	**Weight (%)**	**Heterogeneity, *p*-value**
**EMA**				
Male	2	1.05 (1.01–1.08)	33.72	*I*^2^ = 0.0%, *p* = 0.517
Female	2	1.08 (1.06–1.11)	66.28	*I*^2^ = 24.1%, *p* = 0.251
**NRS**				
Male	3	1.18 (1.11–1.25)	28.38	*I*^2^ = 0.0%, *p* = 0.921
Female	3	1.13 (1.09–1.17)	71.62	*I*^2^ = 33.0%, *p* = 0.225
**DIS**				
Male	4	1.25 (1.10–1.41)	49.38	*I*^2^ = 0.0%, *p* = 0.884
Female	4	1.27 (0.99–1.63)	50.62	*I*^2^ = 71.8%, *p* = 0.014
**DMS**				
Male	4	1.15 (1.05–1.27)	55.96	*I*^2^ = 0.0%, *p* = 0.777
Female	4	1.22 (0.95–1.58)	44.04	*I*^2^ = 70.3%, *p* = 0.117

*EMA, early morning awakening, also referred as trouble waking too early; NRS, non-restorative sleep, also referred as restless sleep or not feeling rested after sleeping; DIS, difficult initiating sleep, also referred as trouble falling asleep; DMS, difficult maintaining sleep, also referred as waking up during the night, trouble waking during night, difficult staying asleep. Forest plot shown in Supplementary Figure 1*.

## Discussion

Insomnia is the most common sleep disturbance globally, which can cause both mental and physical illness. Previous studies have indicated insomnia disorder as a new risk factor of CVDs, other than the traditional atherosclerotic risk factors. However, insomnia patients may complain about different symptoms, including difficulty falling asleep, difficulty maintaining asleep, waking up too early in the morning, and feeling restless after one-night sleep. Patients might have all or just some of these symptoms. It is not clear if all these symptoms had the same association with CVD incidence risk. As a result, this meta-analysis aimed to investigate the relationship between each insomnia symptom and the risk of first-ever CVDs.

Our meta-analysis showed an overall increased risk of CVDs in patients with insomnia symptoms, which was consistent with findings of previous studies. Patients with complaints of DIS (HR 1.22, 95% CI 1.06–1.40), DIM (HR 1.14, 95% CI 1.02–1.27), or NRS (HR 1.16, 95% CI 1.07–1.24) were in higher risk of CVDs individually in this study. Our data indicated that insomnia patients complaining of DIS had much higher risk of CVD than other insomnia symptoms, which was consistent with previous literature. Zheng et al. (13) combined DIS and DMS as one insomnia symptom (DIMS) in a prospective cohort study of 487,200 Chinese adults. The results showed that DIMS was associated with both ischemic heart disease and ischemic stroke but not hemorrhagic stroke (13). Moreover, DIS or NRS was found to be related to 55 or 32% increased risk of CVD mortality, respectively, among 23,447 US men (21). DIS and DMS were related to a higher risk of total strokes (fatal and non-fatal) at first, but this relationship became insignificant after adjusting for age and other risk factors of stroke in the population-based MONICA/KORA Augsburg Cohort Study (15). Our study did not detect a significantly increased risk of CVDs in insomnia patients with EMA symptom. EMA was related to a slight increase in CVD incidence in the study of China Kadoorie Biobank (HR 1.07, 95% CI 1.05–1.09) (13), while the relationship was remarkable neither in male nor female within a Swedish cohort, which consisted of 13,617 subjects ages 45–64 without CVDs at baseline (14).

The mechanisms of increased risk of CVDs in patients with insomnia symptoms are still unclear. The elevated sympathetic and hypothalamic–pituitary–adrenal axis activity is one of the possible mechanisms (22). DIS and DMS in insomnia patients could induce overall short sleep duration, which was proven to be associated with hypercortisolemia and increased catecholaminergic and autonomic activity, resulting in increased blood pressure and impaired glucose metabolism (23–26). High risk of genotype might be another contributing mechanism (27). Furthermore, an experimental study indicated that insomnia disorder could cause abnormal circulating levels of growth hormone, leptin, and ghrelin in younger adults, aggravating the risk of obesity in this population (28). All these factors can lead to an increased risk of CVD incidence.

## Limitations

There were several limitations in the present meta-analysis. Firstly, the insomnia symptoms of enrolled studies were assessed by self-reported questionnaires or interviews. This may lead to a possible higher risk of selection bias due to the interviewers as well as recall and report bias caused by the participants. Some objective instrument (e.g., polysomnography) could be used in future investigations, but the medical expense is still an unsolved problem. Secondly, since we only included studies with clear insomnia symptoms instead of a general conception, this led to the limited number of entered articles compared to previous reviews. However, with the acceptable quality score and large scale of each cohort study and the result of different associations between individual insomnia symptom and CVD risk, our meta-analysis still provided a remarkable reference for future research. Additionally, we could not enter all the included studies in the sensitivity analysis because not all studies provided the separate HR and 95% CI according to male and female. But the primary results showed that there were much more higher heterogeneity across the female subgroup in all insomnia symptoms, especially for DIS and DMS, which showed borderline heterogeneity in the overall analysis. Therefore, female was accepted as the possible origin of heterogeneity and needs to be further investigated in a future study of CVD incidence risk in insomnia patients with different symptoms.

## Conclusions

In conclusion, this meta-analysis indicated that symptoms of DIS, DIM, or NRS were associated with a higher risk of CVD incidence in insomnia patients free of CVDs at baseline. But this association was not significant in insomnia patients complaining of EMA.

## Data Availability Statement

The original contributions presented in the study are included in the article/Supplementary Material, further inquiries can be directed to the corresponding authors.

## Author Contributions

LR and YW conceived and designed the study. TL and SH independently extracted data from the eligible studies and any argument was resolved either by discussion or by involving YW when necessary. SH performed the paper writing with the assistance of TL. All authors contributed to the article and approved the submitted version.

## Conflict of Interest

The authors declare that the research was conducted in the absence of any commercial or financial relationships that could be construed as a potential conflict of interest.
