# Supplementary figures and images for: Individual Insomnia Symptom and Increased Hazard Risk of Cardiocerebral Vascular Diseases: A Meta-Analysis
Source: Front Psychiatry. 2021 May 14;12:654719. doi: 10.3389/fpsyt.2021.654719 (PMC8160242; doi:10.3389/fpsyt.2021.654719)

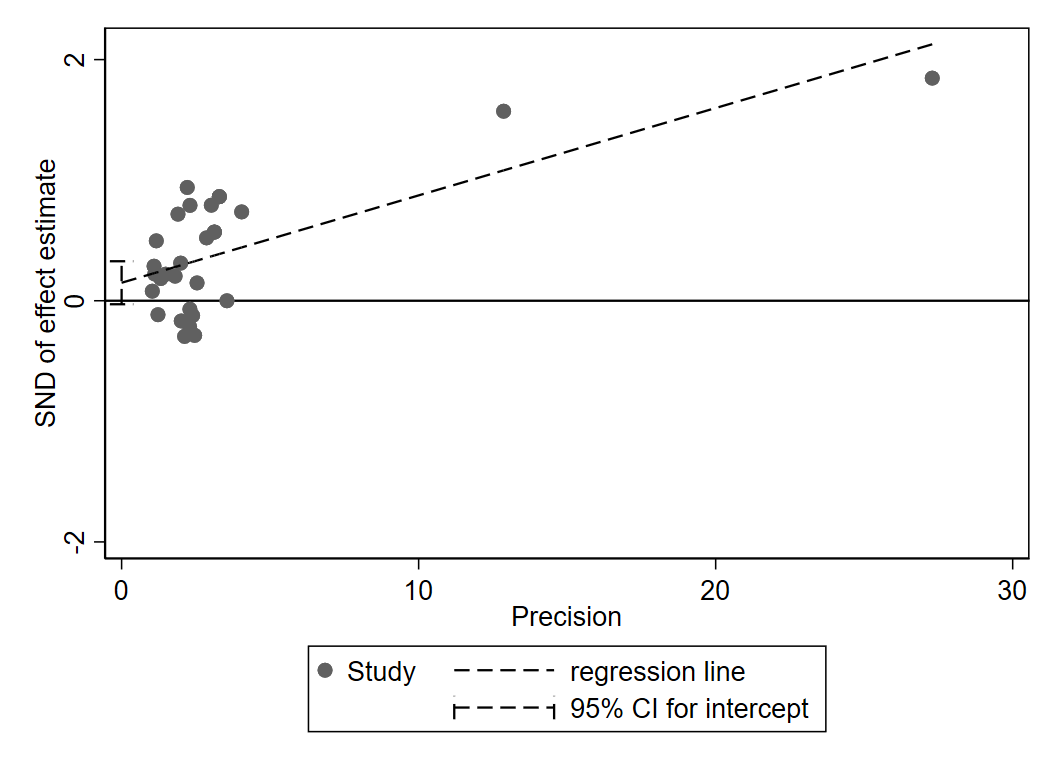

Supplement: Supplementary file 1 [file Image_1.PNG]
